# Very early and severe presentation of Triple A syndrome – case report and review of the literature

**DOI:** 10.3389/fendo.2024.1431383

**Published:** 2024-09-24

**Authors:** Maja Cehic, Katarina Mitrovic, Rade Vukovic, Tatjana Milenkovic, Gordana Kovacevic, Sladjana Todorovic, Sanja Panic Zaric, Dimitrije Cvetkovic, Aleksandra Paripovic, Angela Huebner, Katrin Koehler, Friederike Quitter

**Affiliations:** ^1^ Department of Endocrinology, Mother and Child Health Care Institute of Serbia “Dr Vukan Čupić”, Belgrade, Serbia; ^2^ Department of Pediatrics, Medical Faculty University of Belgrade, Belgrade, Serbia; ^3^ Department of Neurology, Mother and Child Health Care Institute of Serbia “Dr Vukan Čupić”, Belgrade, Serbia; ^4^ Department of Nephrology, Mother and Child Health Care Institute of Serbia “Dr Vukan Čupić”, Belgrade, Serbia; ^5^ Department of Pediatrics, Faculty of Medicine and University, Hospital Carl Gustav Carus, Technische Universität Dresden, Dresden, Germany

**Keywords:** triple A syndrome, TAS, Allgrove syndrome, alacrima, achalasia, adrenal insufficiency

## Abstract

Triple A syndrome (TAS), also known as Allgrove syndrome (OMIM#231550), is a rare, autosomal recessive disorder characterized by the triad of alacrima, achalasia, and adrenal insufficiency. Additional neurological features may be present in two-thirds of patients, involving central, peripheral, and autonomic nervous system manifestations. TAS is caused by genetic alterations in the *AAAS* gene on chromosome 12q13, which encodes the nuclear pore complex protein termed ALADIN (ALacrima, Achalasia, aDrenal Insufficiency, and Neurologic disorder). ALADIN plays a crucial role in nucleocytoplasmic transport of specific proteins, including the transport of DNA repair proteins. TAS exhibits significant phenotypic variability in terms of symptom onset, frequency, and severity, often presenting with a progressive clinical course indicative of an underlying degenerative process. In this study, we report the case of an infant with exceptionally early and severe manifestations of triple A syndrome, with a review of the literature. Our patient exhibited the complete classical triad of TAS at six months of age, being among the youngest reported cases of the syndrome. The clinical course was complicated by severe involvement of the autonomic nervous system, neurogenic bladder, and recurrent urinary tract infections. Subsequently, the patient developed acute pancreatitis, leading to multiorgan dysfunction and a fatal outcome at 25 months of age. This case underscores the potential for atypical disease presentations and the need for clinical awareness in diagnosing and managing patients with TAS.

## 1 Introduction

Triple A syndrome (TAS) or Allgrove syndrome (OMIM#231550) is a rare, autosomal recessive disorder denoted by the triad of alacrima, achalasia, and adrenal insufficiency (1). In addition to these three main characteristics, two-thirds of patients have additional neurological features with central, peripheral, and autonomic nervous system involvement (2–4). Several other features such as xerostomia, dental caries, palmar and plantar hyperkeratosis, dysmorphic facial features, gait disturbances, and delayed puberty have also been associated with this syndrome (2–5).

**Figure 1:**
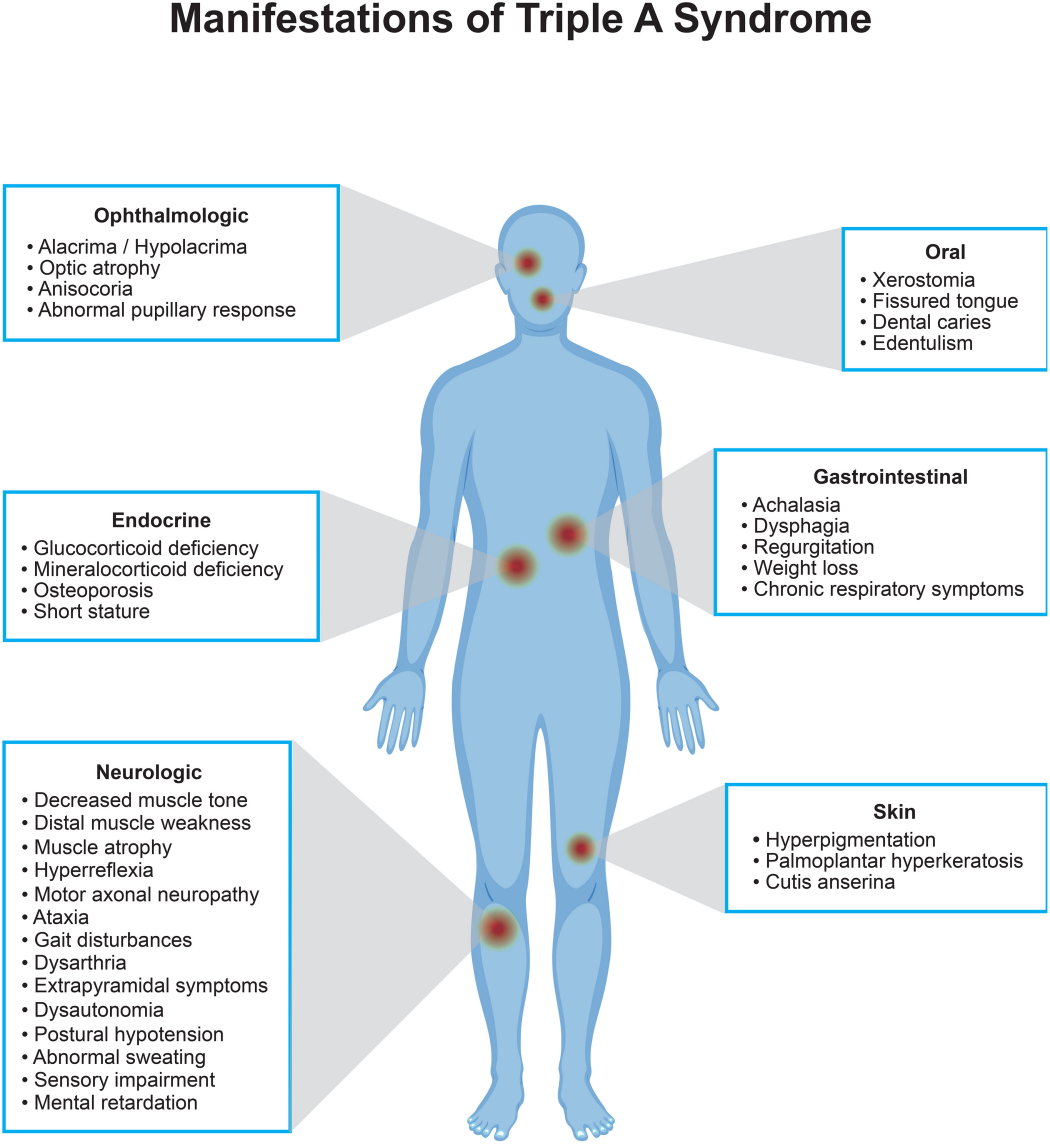
Schematic overview of signs and symptoms of triple A syndrome.

TAS is caused by genetic changes in the *AAAS* gene on chromosome 12q13 (6). This gene encodes the nuclear pore complex protein termed ALADIN (ALacrima, Achalasia, aDrenal Insufficiency, and Neurologic disorder), which regulates the nucleocytoplasmic transport of specific proteins, including DNA repair proteins (7). The *AAAS* gene is ubiquitously expressed in human tissues, which can explain the diversity of symptoms presented in patients, as shown in **Figure 1**. Although the exact pathophysiology is yet to be clarified, in several studies it was shown that mutated ALADIN proteins mislocalize to the cytoplasm, leading to impaired nucleocytoplasmic shuttling of multi-molecular complexes. This makes cells susceptible to oxidative stress which results in selective tissue degeneration (6, 8–11).

In terms of the clinical course, TAS has a high phenotypic heterogeneity regarding the age of onset, occurrence, and severity of symptoms. The onset of clinical features is usually not simultaneous, but rather progressive, suggesting an underlying degenerative process (12). Alacrima is probably the most consistent sign, with a prevalence above 90% in TAS patients, often noted in early infancy (13). Achalasia is present in 75-85%, primary adrenal insufficiency in almost 85%, and the complete clinical triad of TAS in 70% of the cases (3, 13, 14). The first reason for seeking medical help are usually the symptoms of achalasia, whereas adrenal insufficiency often develops gradually during childhood or adulthood.

From a clinical standpoint, the progressive course of symptom development has a protective effect, as once achalasia and/or alacrima is diagnosed in a child, triple A syndrome can be suspected. In these cases, adrenal function is monitored regularly, allowing for the timely diagnosis of adrenal insufficiency. In rare cases when adrenal insufficiency is not heralded by the prior onset of other symptoms, patients are at a high risk of life-threatening adrenal crises. The risk of misdiagnosis here is also high, due to the expectation that other symptoms would precede adrenal insufficiency in TAS, thus omitting TAS from the differential diagnosis of infancy onset adrenal insufficiency. Therefore, it is very important to stay aware of atypical clinical presentations of TAS, especially at an early age.

Herein we present an infant with a very early and severe presentation of triple A syndrome.

## 2 Case presentation

An 11-month-old male infant was admitted to our hospital for evaluation of muscle hypotonia and developmental delay. Parents were nonconsanguineous and the pregnancy was uneventful. His birth weight and length were within the normal range, with a birth weight of 4050 grams and a birth length of 54 centimeters. Decreased muscle tone, feeding problems, vomiting, and weight loss were noticed since six months of age. Two months prior to admission the boy suffered from repeated urinary tract infections leading to recurrent fever. On clinical examination the child showed failure to thrive (weight 6040 g, Z score -4,09 SD; length 75,0 cm, Z score -0,02 SD), pale skin with hyperpigmentation in the lumbar region, abdominal distension without organomegaly, and generalized muscle weakness. Upon further questioning, parents recalled that the infant cried without tears since birth.

### 2.1 Endocrine investigations and management

Baseline blood investigations revealed normal complete blood count and creatinine. Electrolyte levels indicated salt wasting (serum Na 125 mmol/L, K 6.2 mmol/L, urine Na 30 mmol/L). Chest radiography showed signs of previous repeated aspiration pneumonia. Abdominal ultrasound examination was unremarkable. In the light of clinical presentation and initial laboratory results, the clinical diagnosis of TAS was suspected. Further investigations revealed elevated morning ACTH of 886 pg/mL (normal range < 46 pg/mL), with borderline low serum cortisol of 165 nmol/l (normal range 101-536 nmol/L). Renin level was elevated at > 500 µIU/mL (normal range 2,8 – 39,9 µIU/mL), while aldosterone levels were within the normal range at 40,6 ng/dL (6,5-86 ng/dL). Despite the typical constellation of primary adrenal insufficiency in the laboratory results, an ACTH test was performed [max. cortisol concentration of 159 nmol/L after 60^th^ minute, 17-hydroxyprogesterone (17-OHP) levels were low throughout the test (0,7 – 0,9 nmol/L)]. Diagnosis of primary adrenal insufficiency was established, and hydrocortisone and fludrocortisone therapy were started.

### 2.2 Additional evaluations and genetic testing

Ophthalmic evaluation revealed reduced tear production consistent with alacrima, and local therapy with artificial tears was introduced. Due to feeding difficulties and repeated vomiting, a barium swallow was performed, revealing severe stenosis of the lower esophageal sphincter and dilatation of the upper esophagus. The diagnosis of achalasia of the cardia was established and managed with esophagomyotomy at the age of 19 months.

Molecular genetic testing confirmed a compound heterozygous mutation in the *AAAS* gene consisting of a T>C transition in exon 8 (c.787T>C, p.Ser263Pro; paternal) and a C>T transition in exon 12 (c.1159C>T, p.Gln387*; maternal), thus confirming the diagnosis of the triple A syndrome.

### 2.3 Neurological progression and further clinical course

At 17 months of age, the patient was admitted to the nephrology department due to repeated urinary tract infections. After establishing the diagnosis of neurogenic bladder, intermittent bladder catheterization was introduced, and antibiotic prophylaxis started. At the age of 22 months, the patient presented with intermittent fever over the course of twenty days, hypoalbuminemia and nephrotic range proteinuria. Kidney biopsy was performed, pathohistological examination revealed mesangioproliferative glomerulonephritis, and immunosuppressive therapy with cyclosporine and tacrolimus was initiated.

Transitory left arm paresis occurred at the age of 23 months. CT scan of the head showed a moderate reduction of white matter volume, with *ex vacuo* supratentorial ventricular system expansion. In later course, the patient presented with focal seizures in the form of left-arm spasms and ipsilateral eyelid twitching. Video electroencephalography showed continuous epileptiform discharge activity, and levetiracetam was started. At the age of 25 months balloon dilatation of the esophageal stricture was performed. After the procedure, the patient developed clinical and laboratory signs of sepsis. Chest radiography showed signs of pulmonary congestion. The further clinical course was complicated by acute pancreatitis, with multiorgan dysfunction and unfortunately a fatal outcome at 25 months of age.

## 3 Discussion and review of the literature

Triple A syndrome is a very rare disorder with an estimated prevalence of 1 in 1 million individuals, though it is hypothesized to be higher due to missed diagnoses (15). Early diagnosis may present a challenge, given its rarity and high phenotypic heterogeneity, even within families. The syndrome generally manifests during the first two decades of life, with the mean age of onset and diagnosis being around five years of age, with a high rate of sudden deaths due to acute adrenal crisis if the disease is not recognized timely (4, 13, 16).

### 3.1 Alacrima

Alacrima or hypolacrima is the most common first symptom of TAS, being present in 90-100% of reported cases (13, 17, 18). In most patients, it is usually noticed by parents during infancy. Still, its significance is usually overlooked, as it does not always prompt parents to seek professional help, and is recognized as a part of triple A syndrome only in retrospect in many cases (13, 15).

Autonomic nervous system (ANS) dysfunction at the level of lacrimal glands has been suggested as the cause of the failure of tear production, and may further lead to corneal destruction (19). The gold standard of diagnosis is the Schirmer test, and management is symptomatic, using artificial tears and eye drops.

Alacrima itself is a rare condition, and it has only been described in a limited number of congenital disorders. Careful history taking, clinical and laboratory examinations should lead to an underlying cause (20). Hence, in a patient presenting with adrenal insufficiency or achalasia, the presence of alacrima can point toward the diagnosis of TAS, whereas its absence almost rules out this condition (13).

Similar to the majority of reported cases, our patient had alacrima since the earliest infancy, which was not recognized until the direct questioning of parents about this specific symptom. However, early onset and severe presentation of other TAS components is what sets this patient apart and emphasizes the need for continuous education of physicians, to increase awareness regarding the atypical presentations of TAS.

### 3.2 Achalasia

Achalasia represents a primary motor disorder of the esophagus, characterized by lower esophageal sphincter function impairment, and a loss of esophageal peristalsis due to an imbalance between excitatory and inhibitory neurons (21). With a prevalence of over 90%, it is the second most common disorder in patients with TAS (13). It usually develops in mid-childhood between three months to 16 years of age, and is typically the first symptom for seeking medical advice (13, 22). Swallowing difficulty, vomiting, chronic cough, dyspnea, and weight loss may precede the diagnosis of achalasia years before it being suspected. Diagnosis is supported by a barium swallow study and esophageal manometry results. Multimodal therapy consists of pharmacological treatment with nifedipine, pneumatic dilatation, laparoscopic Heller myotomy, and peroral endoscopic myotomy (23). However, achalasia progression can be severe, with a high rate of treatment failure (24).

Our patient presented symptoms of achalasia with vomiting and failure to thrive since the earliest infancy. Despite surgical treatment by Heller myotomy, he had esophageal restenosis, which required repeated pneumatic dilatations.

### 3.3 Adrenal insufficiency

In the majority of individuals with TAS, adrenal insufficiency (AI) manifests during the first decade of life, although rarely it can remain undiagnosed until 50 years of age (22). According to a systematic review of the literature, the median age of onset of AI in patients with TAS is four years (range 0 – 23 years) (13).

In most cases it manifests acutely as adrenal crisis with hypoglycemia and hypotension, thus being the leading cause of mortality in undiagnosed TAS patients (15, 25). Diagnosis is confirmed by a low morning cortisol and an absent cortisol response during the Synacthen (ACTH) test. While the ACTH test is considered the gold standard for confirming the diagnosis, in cases with extremely high ACTH levels, and low sodium and cortisol levels, performing the test is not essential for establishing the diagnosis of adrenal insufficiency. It is important to note that the evaluation of adrenal function in TAS patients should not be limited to glucocorticoid deficiency, since associated mineralocorticoid deficiency is reported in up to 15% of these patients (26). Due to the degenerative nature of the disease, the possibility of mineralocorticoid deficiency developing later on cannot be excluded, even if it is not present initially. The replacement therapy with hydrocortisone is always indicated in patients with overt glucocorticoid deficiency; and in some cases, fludrocortisone as well.

Suspecting triple A syndrome, repeated evaluation of adrenal function is of high importance during follow-up. In some patients, adrenal gland dysfunction may be mild, subclinical, or even absent during the first two decades of life, making the early diagnosis of adrenal insufficiency, family education, and institution of substitution therapy crucial for the prevention of adrenal crises (3). This is in most patients facilitated by the fact that the diagnosis of TAS has already been suspected or established due to the prior onset of alacrima and/or achalasia. Although there are no specific guidelines for the surveillance of patients with TAS, lifelong follow-up is essential. Apart from history taking and physical exam, endocrine monitoring regarding adrenal function should include blood pressure monitoring, glycemia, serum electrolytes, cortisol, ACTH, DHEA-S, renin, and aldosterone levels.

Biannual monitoring should be sufficient for patients who do not have adrenal insufficiency at the time of diagnosis, with more frequent check-ups if suggestive symptoms or signs develop. DHEAS can serve as an effective marker for assessing zona reticularis function after the usual age when adrenarche occurs, facilitating the detection of glucocorticoid insufficiency.

Upon the diagnosis of adrenal insufficiency, more frequent monitoring is required - initially, every 3-6 months, and once stable, a biannual monitoring should be sufficient.

Regular follow-ups and adjustments to the monitoring plan are necessary, particularly during periods of stress, illness, or surgery, as these can precipitate adrenal crises. This proactive approach helps in the timely management and prevention of complications associated with TAS. In the current literature, there are several reported cases of adrenal insufficiency presenting in infancy and later to be diagnosed with TAS. To the best of our knowledge, this is the first detailed case report of a patient displaying the full clinical picture of adrenal insufficiency at 11 months of age, which highlights the need for being aware of atypical clinical presentations of TAS (27, 28).

### 3.4 Neurological symptoms, developmental delay, and other symptoms

Neurological symptoms present in approximately 60% of the TAS patients, featuring peripheral neuropathy, autonomic impairment, pyramidal and bulbar dysfunction, and cerebellar and neuroophthalmological symptoms (26, 29). Other neurological symptoms include distal weakness and atrophy, mixed sensory-motor demyelinating neuropathy, intention tremors, gait imbalance, motor neuron disease, and optic atrophy (15, 30–32). Developmental delay presents a significant cause of concern for the parents. Though cognitive impairment may not be present in all cases, it has been reported in many children with Triple A syndrome. Apart from focal demyelination of the medulla and inferior cerebellar peduncle, no specific finding in brain magnetic resonance imaging has been described in the literature (33).

The age of onset of neurological symptoms varies, ranging from 2 to 25 years (14). According to published data, signs of neurological dysfunction are usually not present at the time of diagnosis. However, in rare cases, it may be a presenting symptom in childhood (3). Our patient presented with signs of hypotonia and neurological deterioration since six months of age, which is significantly earlier than in most described cases. Repeated urinary tract infections in the presented patient were most likely a consequence of autonomous nervous system involvement with neurogenic bladder. Having in mind that there are no described cases of this type of ANS involvement in patients with TAS, the neurogenic bladder with repeated urinary tract infections presents a novel ANS symptom newly described in our patient, highlighting the need for detailed neurological and nephrological assessment of these patients at diagnosis. According to the published data, to the best of our knowledge, there has been no correlation of mesangioproliferative glomerulonephritis (MPGN) in TAS patients so far. We, therefore, assume that the mesangioproliferative glomerulonephritis seems to be a coincidental disease in our patient.

Although neurological aspects of the syndrome are progressive and hardly treatable, a new possible approach with N-acetylcysteine has been recently reported in a single patient (11).

In the mouse model, the lack of ALADIN leads to infertility in females suggesting that the protein exhibits as yet unknown effects on the meiosis and maturation process of oocytes (34). However, clinical data on puberty and fertility in patients are scarce. There is only one reported case of a female patient with homozygous *AAAS* mutation who successfully conceived and delivered a baby (35). Additionally, the same study reported on six male patients with TAS who all entered puberty on time, with normal plasma levels of gonadotropins, testosterone, inhibin B, AMH and low levels of adrenal androgens (35). There have also been isolated reports of delayed puberty in two cases (5, 36).

Although limited, available data indicate that patients with TAS exhibit low levels of DHEAS and lack adrenarche (14, 37). This suggests that the progressive degeneration of the zona reticularis is associated with ceasing steroid production in zona fasciculata. The adrenal zona reticularis may be particularly vulnerable to disrupted redox balance and oxidative damage. Low DHEAS levels lead to decreased libido, sexual dysfunction, and other features of “adrenopause” in early adulthood. DHEA supplementation should be considered to enhance these patients’ health and well-being.

Short stature and cachexia are common in TAS, with poor growth observed in up to 30% of patients diagnosed with TAS regardless of the presence of adrenal insufficiency (unpublished data).

### 3.5 Pathogenetic mechanisms and clinical course variability

The phenotype of TAS is complex and clinical features are progressive, with the exact pathophysiological mechanisms involved in the disease remaining unclear. Clinical features of adrenal insufficiency and neurodegeneration affecting the central, peripheral, and autonomic nervous systems are not present at birth but rather develop over time, implicating that a progressive degenerative process is involved in the pathogenesis of this disorder. In some cases, a delay in diagnosis may occur due to paucisymptomatic manifestations during childhood and slow disease progression (38–40).

While the exact function of the ALADIN protein remains mostly unclear, there have been attempts to clarify the pathogenetic mechanisms of the disease.

Increased oxidative stress has been shown to lead to apoptosis and tissue damage in patients with triple A syndrome, and several *in vitro* studies have documented oxidative stress in cultured adrenal, neuronal cells, and fibroblasts harboring *AAAS* mutations (8, 10, 12, 41). In patients’ fibroblasts, altered induction, or downregulation of genes associated with oxidative stress and antioxidant defense have been shown (9). Furthermore, ALADIN interacts with the ferritin heavy chain protein (FTH1), which, in addition to its iron storage role, protects the nucleus from oxidative damage (41). In an *in vitro* study, strong evidence was provided that *AAAS* knock-down in a human adrenocortical tumor cell line results in significant functional impairment of steroidogenesis, especially in the glucocorticoid and androgenic pathways, as well as an impairment in the cellular response to oxidative stress (42). One possible explanation for the preservation of the zona glomerulosa in the majority of the patients could be the lower production of reactive oxygen species during aldosterone synthesis, in contrast to the cortisol synthesis in the zona fasciculata (42).

The *AAAS* gene is ubiquitously expressed in human tissues, with predominance in the adrenal and pituitary glands, and central nervous system. Although this may explain the susceptibility of the adrenal gland and nervous system, pituitary dysfunction is not typically associated with the syndrome. However, there has been a reported case of a patient with growth hormone deficiency (43).

In one study it was suggested that antioxidant treatments may prove to be a viable therapeutic strategy to slow down, or even stop the progression of triple A syndrome disease (12). In an *in vivo* study it was demonstrated that the use of N-acetyl cysteine in the treatment of a boy with triple A syndrome reduced reactive oxygen species. However, the long-term effect of treatment with NAC should be evaluated to determine its real benefits in the prevention of degenerative processes in triple A syndrome (11).

Intra-familial variability in the clinical phenotype of patients with TAS is well recognized. Studies of genotype-phenotype correlation in TAS patients revealed the absence of consistency, proposed to be due to the effect of other modifying genes or environmental factors on the phenotype of TAS (4, 44). **Table 1** lists all the published cases of early diagnosed TAS, illustrating the high phenotypic variability of this syndrome.

**Table 1:** Published cases of early diagnosed Triple A syndrome patients.

Author	Year	Patients, n	Age at presentation (years, mean)	Symptoms at presentation	Alacrimia (age of presentation; mean)	Achalasia (age of presentation; mean)	Adrenal insufficiency (age of presentation; mean)	Neurological symptoms (age of presentation; mean)	Other manifestations
Grant, et al. (26)	1993	20			19/20 (early infancy)	15/20 (0.5 – 16 years)	20/20 (1.0 – 8.3 years)	17/20	11 patients had autonomic dysfunction
Gazarian, et al. (29)	1995	4	3 – 4.5 years	Seizures, vomiting, lethargy,	4/4	3/4	3/4	4/4	Short stature, dysmorphic facial features
Moshe, et al. (45)	1996	2	1,8 – 4 years	Hypoglycemia, vomiting,	Yes	Yes (diagnosed at 1,8 and 2 years)	1/2 (diagnosed at the age of 4)		
Prpic, et al. (4)	2003	3	1.8 – 10 years	Vomiting, dysphagia	2/3	3/3	2/3	3/3	Autonomic dysfunction, skin changes
Brooks, et al. (19)	2004	1	4 years		Yes	Yes (2 years of age)	Yes (1 year of age)	Yes	Dry tongue, palmoplantar hyperkeratosis
Milenkovic, et al. (36)	2008	3	3.5 – 12 years	Vomiting, dysphagia, skin hyperpigmentations,	3/3 (NB)	2/3 (1.5 – 6 years)	1/3 (7 years)	2/3	
Toromanovic, et al. (46)	2008	1	5.8	Skin hyperpigmentations, vomiting, weakness, fatigue, leg pain	Yes (NB)	Yes (10 months)	Yes (5.8 years)	Yes	Dysmorphia,
Kind, et al. (10)	2010	13	1 – 8 years		12/13	10/13	10/13	12/13	Mental retardation, ps cavus, visual problems
Milenkovic, et al. (3)	2010	10	1.2-9.4	Fatigue, skin hyperpigmentations, hypoglycemia, seizures, dysphagia, vomiting, failure to thrive	10/10 (0 – 8.6 years)	7/10 (0.8 – 14 years)	8/10 (5.8 – 9.4 years)	8/10 (1.5 – 9.4 years)	Dysmorphic facial features, short stature
Alhussaini, et al. (24)	2011	9	2 – 11 years (mean age 7,1 years)	Vomiting, dysphagia, regurgitation, weight loss, chronic cough, peripheral neuropathy	9/9	9/9	7/9		Autonomic dysfunction in 6 patients
Dumic, et al. (2)	2012	8	2 – 11 years	Dysphagia, vomiting, failure to thrive, seizures, hypoglycemia, skin hyperpigmentations	8/8	5/8	7/8	6/8	Postural hypotension, mental retardation, palmoplantar hyperkeratosis
Kallabi, et al. (18)	2015	26	1.1 -10 years (mean age 2 years and 9 months)	NA	26/26	23/26	26/26	7/26	
Eringel, et al. (47)	2016	3	1.3 – 17 years	Vomiting	3/3	3/3 (infancy – 17 years)	3/3 (1-5 years)		
Fragoso, et al. (11)	2016	1		Jaundice, severe anemia, developmental delay, repeated otitis	Yes	Yes (0.5 years)	+ (at 3 years of age)	Yes	Short stature
Kurnaz, et al. (16)	2017	6	3 – 7.5	Fever, seizures, vomiting, diarrhea	6/6	4/6	6/6	3/6	
Patt, et al. (13)	2017	8	4-10 years		8/8 (early infancy)	7/8 (3-20 years)	7/8 (2,5 – 10 years)	4/8	
Tibussek, et al. (20)	2017	2	0,7-3,4 (2,1)	Vomiting, regurgitation, recurrent respiratory tract infections, hypoglycemic myoclonic events, anisocoria	Yes	Confirmed in one patient	Confirmed in one patient	Present in both patients	Frequent respiratory tract infections in infancy
Singh, et al. (27)	2018	7	0.3 – 12 years	Developmental delay, skin hyperpigmentations, failure to thrive, gait imbalance, hypoglycemia	7/7 (NB)	7/7 (3 – 15 years)	5/7	4/7	
Berrani, et al. (48)	2018	2	1 – 2.6 year	Anorexia, vomiting, skin hyperpigmentation, failure to thrive, generalized weakness	2/2 (NB)	2/2 (1- 1.3 years)	2/2 (1 – 1.3 years)		Autonomic dysfunction
Lu, et al. (49)	2019	1	3 years	Loss of consciousness, hypoglycemia	Yes (3 years)	Yes (9 years)	Yes (3 years)	Migraines (age of 9)	
Polat, et al. (37)	2019	23	1,3-18,5	Hypoglycemic seizures, skin hyperpigmentation, swallowing difficulty, vomiting, failure to thrive	In all patients 23/23 (NB)	13/23 (1,75 – 13,8 years, median age 10 years)	18/23 (1,3-13 years, median age 3,4 years)	18/23	Dental abnormalities, short stature, palmoplantar hyperkeratosis, orthostatic hypotension,
Ain, et al. (50)	2019	1	1 year	Vomiting, skin hyperpigmentations, swallowing difficulty, fainting	Yes (diagnosed at the age of 3)	Yes (diagnosed at the age of 3)	Yes (diagnosed at the age of 3)	Hyperreflexia (3 years)	
Gupta and Dayal (51)	2020	1	2,5 years	Progressive darkening of lips, absence of tears	Yes (NB)	No	Yes (2.1 years of age)	No	
Rivera-Suazo, et al. (52)	2021	1	0.8 (diagnosis at 1,8 years)	Malnutrition, vomiting, dysphagia	Yes (NB)	Yes (9 months at presentation)	No		
AlOmran, et al. (28)	2021	1	0.9 years	Vomiting, lethargy	Yes	No	Yes		

NA, not available, NB, newborn.

## 4 Conclusion

A wide spectrum of clinical conditions comprising TAS requires a multidisciplinary approach to the disease, involving different clinical disciplines for the diagnosis, follow-up, and therapeutic decisions. It is of great importance to exhibit a high index of suspicion for the diagnosis of TAS, even in patients presenting only with isolated components of the syndrome (22). Given the rarity and high phenotypic heterogeneity, early identification of the triple A syndrome is challenging. According to the published data, the mean age of onset of the first symptom is between 1 and 13 years (14, 22). Our patient was six months old when he presented the complete triad of classical TAS components, being among the youngest children with triple A syndrome reported so far. Additionally, our patient had severe ANS involvement with neurogenic bladder and repeated urinary tract infections, previously not described in the literature and highlighting the possibility of atypical disease presentation.
